# Prominent crista terminalis mimicking a right atrial mass: A case report and brief review of the literature

**DOI:** 10.1016/j.radcr.2021.11.028

**Published:** 2021-12-09

**Authors:** Dhairya A Lakhani, Aneri B Balar, Cathy Kim

**Affiliations:** Department of Radiology, West Virginia University, Morgantown 26506, WV

**Keywords:** Cardiac mass, Crista terminalis, Right atrial mass, Echocardiogram

## Abstract

The crista terminalis is a normal anatomical structure, characterized by a smooth muscular ridge along the superior aspect of the right atrium. It is derived from resorption of the right valve of the sinus venosus and it divides the right atrium into smooth posteromedial and trabeculated anterolateral portions. Crista terminalis is not normally detected in the standard views of transthoracic echocardiogram and non-gated CT of the chest. In rare circumstances, the crista terminalis may be prominent and could lead to misdiagnosis as a malignant process, such as in our case. A comprehensive understanding of the crista terminalis anatomy, and its characteristic appearance on transthoracic echocardiogram, CT and PET/CT will minimize the risk of misdiagnosis and will avoid patient anxiety with more extensive examinations. Here, we present a case of a 78-year-old male with newly diagnosed high-grade invasive urinary bladder urothelial carcinoma. Pre-operative transthoracic echocardiogram reported as 2 cm right atrial mass concerning a metastasis lesion. Subsequent evaluation with MRI cardiac morphology confirmed the diagnosis of benign prominent crista terminalis, a normal anatomical structure.

## Background

Right atrium is comprised of two portions, the “smooth” portion and heavily trabeculated anterolateral portion. The “smooth” portion is derived from embryologic sinus and is known as sinus venarum. A smooth fibromuscular ridge along the superior aspect of the right atrium divides the right atrium into smooth sinus venarum and trabeculated right atrium, this ridge is known as crista terminalis or terminal crest [1,2]. Crista terminalis is a horseshoe or twisted C-shaped fibromuscular ridge that originates from inter-atrial septum, extends anteriorly towards the right of superior vena cava orifice, descends along the posterolateral wall of the right atrium, turns anteriorly and terminates right to the inferior vena cava orifice. It represents the remnant tissue from the septum spurium. Septum spurium divides the embryological primitive right atrium and sinus venosus [1], [2], [3].

There is wide variation in size of the crista terminalis, depending on the degree of regression of septum spurium [3,4]. Crista terminalis is an important landmark in conduction of heart rhythm, owing to its relationship to sinoatrial node and vessels. It may be the site of origin for tachyarrhythmias and is often a target for radiofrequency ablation [5,6]. Crista terminalis is present in every heart, but not always seen on imaging. The size of crista terminalis have been reported in literature ranging from 3 mm to 6 mm. It is more prominent superiorly. There is no consensus on a normal size [3,7]. A prominent crista terminalis is reported in about 40% of the patients, some cases measuring up to 15 mm, which may lead to misdiagnosis as malignancy [3,7,8].

Prominent Crista terminalis may incidentally be detected on Echocardiogram, best visualized in apical four chamber view [1]. The characteristic imaging features of Crista terminalis on transthoracic echocardiogram includes: (i) Location: Along the posterolateral wall of right atrium near the superior vena cava, along the course of crista terminalis connecting the both vena cavae; (ii) Smooth, round margins; (iii) Isoechoic to adjacent myocardium; (iv) Phasic change in size, becoming thicker or larger during atrial systole. These features can help in distinguishing it from the right atrial tumor or thrombus [1,3]. Externally, crista terminalis corresponds to the sulcus terminalis. Internally crista terminalis extends from the superior vena cava to inferior vena cava, along the lateral right atrial wall [1]. Transesophageal echocardiogram in bicaval view allows simultaneous visualization of the venae cavae, atria, and interatrial septum [3].

Presence of Crista terminalis is of no clinical significance, in some occasions, it is associated with atrial tachyarrhythmias [1,2,8].

Here, we present a case of a 78-year-old male with newly diagnosed high-grade invasive urinary bladder urothelial carcinoma, with prominent crista terminalis on pre-operative transthoracic echocardiogram, initially reported as 2 cm right atrial mass concerning metastasis. Subsequent evaluation with MRI cardiac morphology confirmed the diagnosis of prominent crista terminalis, a normal anatomical structure.

## Case report

**Fig. 1:**
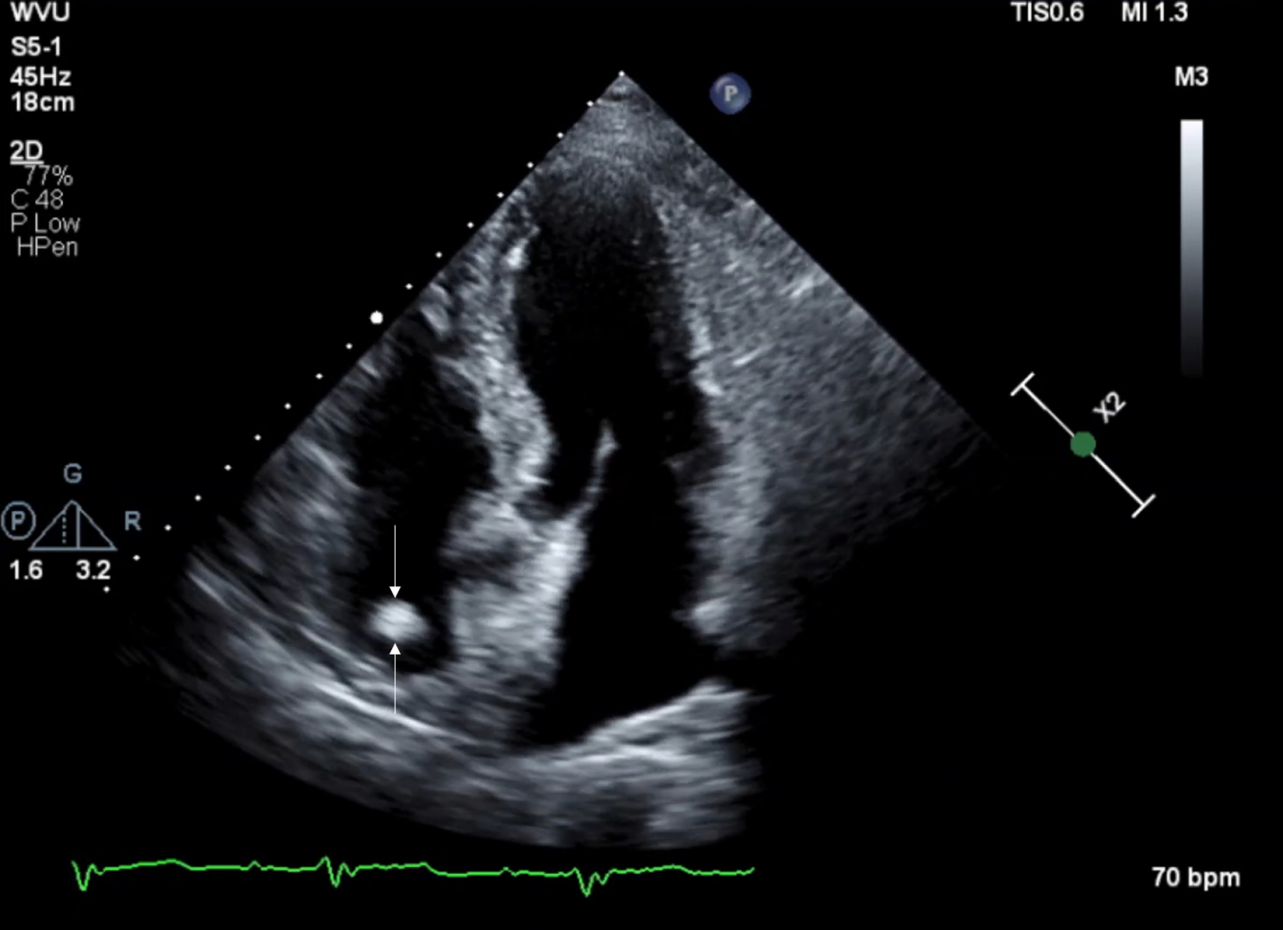
Figure 1: Transthoracic echocardiogram. Transthoracic echocardiogram was performed as a part of preoperative workup prior to surgery. Four-chamber transthoracic echocardiogram shows a prominent crista terminalis (arrow) in the right atrium, which was initially reported as indeterminate, and concerning for malignancy. Further evaluation with CT or MRI cardiac morphology was recommended

A 78-year-old male with newly diagnosed bladder cancer was undergoing transthoracic echocardiogram as a part of preoperative workup. Four-chamber transthoracic echocardiogram (Fig. 1 and supplemental video 1) shows a prominent crista terminalis (arrow) in the right atrium, which was initially reported as indeterminate, and concerning for malignancy. Further evaluation with CT or MRI cardiac morphology was recommended.

**Fig. 2:**
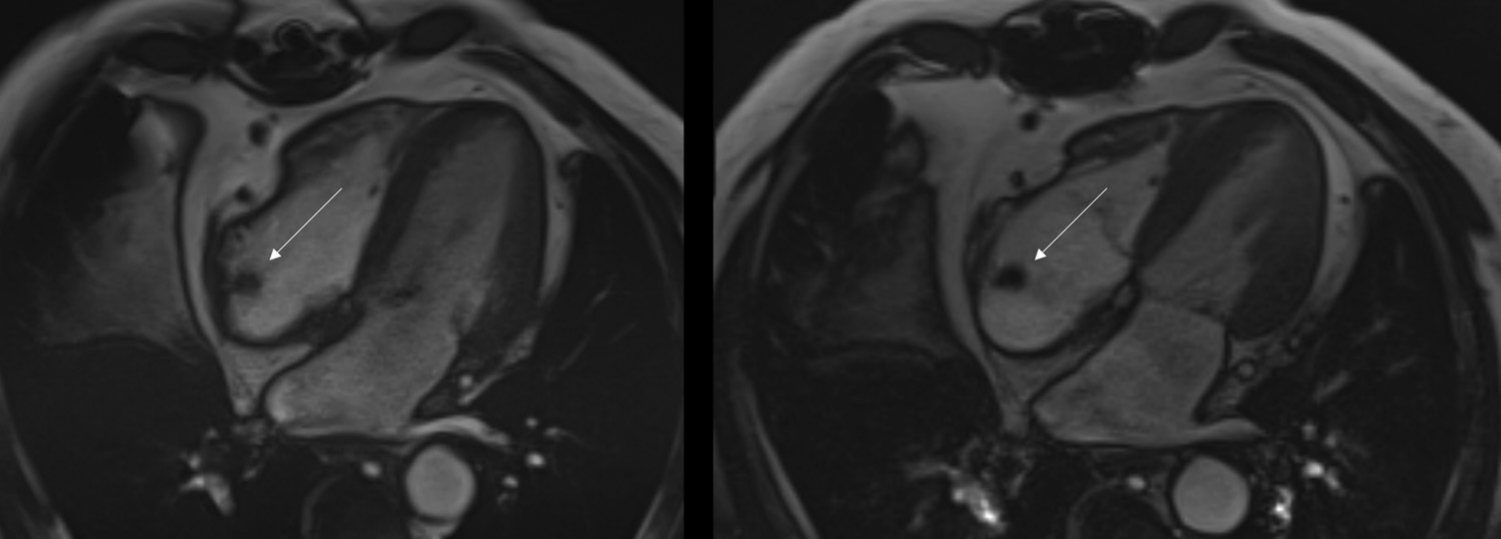
Figure 2: Cardiac MRI without contrast. Further, evaluation with MRI cardiac morphology was performed. The study was limited due to motion and refusal to administer contrast. TRUFI (true fast imaging with steady-state free precession) axial images in four-chamber view demonstrates a prominent right atrial intracavitary lesion

**Fig. 3:**
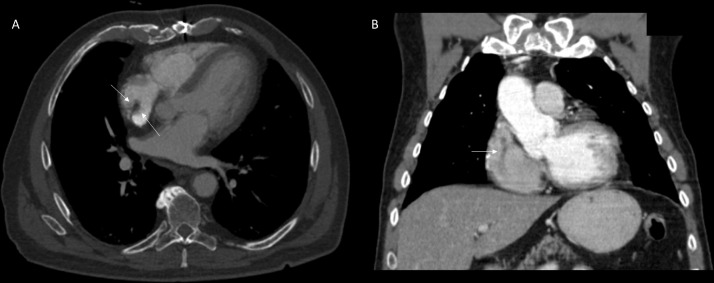
Figure 3: PET/CT examination. MRI cardiac morphology when compared to prior CT component of the PET/CT examination showed classic characteristic findings of crista terminalis. Axial (3A) and Coronal (3B) images show crista terminalis along the lateral wall of the Right atrium. Coronal image shows the complete craniocaudal extent of the crista terminalis (arrow)

**Fig. 4:**
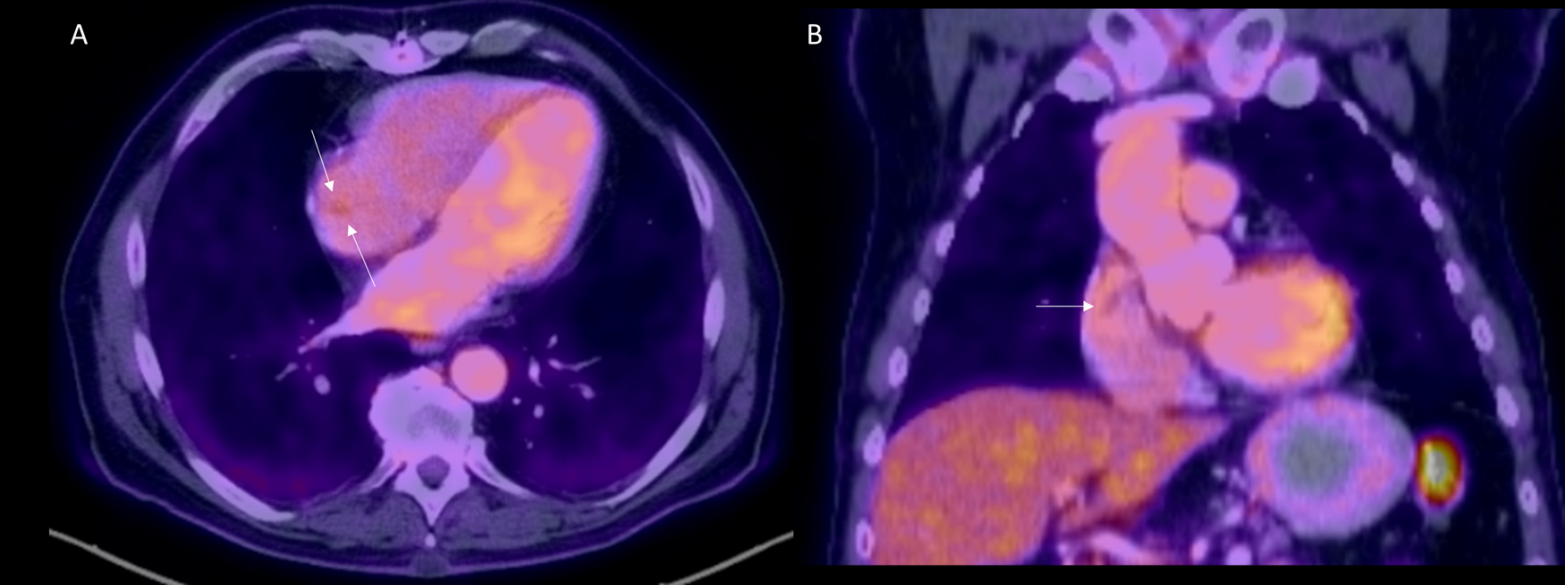
Figure 4: PET/CT examination: MRI cardiac morphology when compared to prior PET/CT had classic characteristic findings of crista terminalis. Axial (4A) and Coronal (4B) images show crista terminalis along the lateral wall of the Right atrium. Coronal image shows the complete craniocaudal extent of the crista terminalis (arrow). No abnormal metabolic activity was appreciated on the PET/CT component of the examination

MRI cardiac morphology was performed (Fig. 2 and supplemental video 2). The study was limited due to motion and refusal to administer contrast. TRUFI (true fast imaging with steady-state free precession) images (Video 1 and Fig. 2) in four-chamber view demonstrates a prominent right atrial intracavitary lesion. Which when compared to prior PET/CT had classic characteristic findings of crista terminalis (Fig. 3, Fig. 4). Axial (3A and 4A) and Coronal (3B and 4B) images show crista terminalis along the lateral wall of the Right atrium. Coronal image shows the complete craniocaudal extent of the crista terminalis (arrow). No abnormal metabolic activity was appreciated on the PET/CT component of the examination.

No further cardiac or imaging workup was recommended.

## Discussion

Owing to technological advances, imaging is now an integral component in evaluation of cardiovascular disorders. Multimodality approach including echocardiography, CT, MRI, and invasive angiography are used in evaluating cardiovascular disorders [3]. The improved spatial and temporal resolution of imaging modalities have led to increased detection of normal anatomical variants and structures, which may lead to misdiagnosis. Hence, a comprehensive understanding of normal anatomical variants, and its characteristic appearance on transthoracic echocardiogram, CT and PET/CT is essential [1,3,[9], [10], [11], [12], [13], [14]].

The normal anatomical structures and anatomical variants in heart, which presents as band or band-like structures includes: Crista terminalis, taenia sagittalis, chiari network, coumadin ridge, moderator band, papillary muscles, and chordae tendineae [1,3]. A multi-modality imaging approach is typically used in characterization of these structures and differentiating them from pathology. Typical indication for further imaging includes incidentally detected variants of unclear etiology, incompletely characterized and/or incompletely evaluated on echocardiogram [1], [2], [3].

Transthoracic echocardiogram is the first-line imaging modality for evaluation of intracavitary cardiac lesions. It is widely available, portable, cost-efficient and safe. The limitations include subjective variations in interpretation, narrow field-of-view, limited tissue characterization and acoustic window in patients with obesity, chest wall deformity or COPD. The second-line imaging modality includes Cardiac CT and MRI. The advantages of cardiac CT includes wide availablility, rapid turnaround time, high spatial and temporal resolution, superior anatomical depiction, wide field-of-view and multiplanar reconstruction capabilities. The limitations of cardiac CT includes associated radiation dose, iodinated contrast administration, and lower efficacyin patients with arrythmias and hemodynamic instability. The advantages of cardiac MRI includes good spatial and temporal resolution, excellent depiction of anatomy, contrast resolution and tissue contrast, wide field-of-view and reconstruction capabilities. The limitations of cardiac MRI includes higher cost, longer scan time, not widely available, requires sinus rhythm, gadolinium associated adverse effects and can not be used in patients with non-MRI compatible hardware and devices [1,3,[15], [16], [17], [18], [19], [20]].

Cardiac MRI is the preferred modality for evaluation of indeterminate intracavitary lesions. Tissue characterization is one of the major strengths of cardiac MRI [1,3,[15], [16], [17], [18]]. The key factor to differentiate normal anatomical variant from pathological lesion on MRI includes evaluation of signal intensity and enhancement characteristics in comparison with adjacent normal myocardium. The normal anatomical variants would demonstrate a similar enhancement pattern and would be isointense to normal myocardium. Where-as pathological lesions will have altered signal intensity and enhancement characteristics compared to adjacent myocardium [1,3,[15], [16], [17], [18]].

Cardiac CT can also be used for characterization of indeterminate intracavitary lesions, however, it is typically reserved for cases where cardiac MRI is not possible. CT provides limited tissue characterization. Hence, normal variants are typically distinguished from masses based on their location and ancillary features (including: attenuation values, contrast enhancement and local invasion). Delayed phase (45–60 seconds) can help in differentiating intracavitary thrombus from normal variants [9,15,16,19,20]. Thrombus would remain low attenuating, whereas normal variants will show contrast enhancement similar to adjacent myocardium [1,3,9].

## Appendix Supplementary materials

*Supplemental video 1***:** Transthoracic echocardiogram was performed as a part of preoperative workup prior to surgery. Four-chamber transthoracic echocardiogram shows a prominent crista terminalis (arrow) in the right atrium, which was initially reported as indeterminate, and concerning for malignancy. Further evaluation with CT or MRI cardiac morphology was recommended. *Supplemental video 2***:** Further, evaluation with MRI cardiac morphology was performed. The study was limited due to motion and refusal to administer contrast. TRUFI (true fast imaging with steady-state free precession) Cine images in four-chamber view demonstrates a prominent right atrial intracavitary lesion. VIDEO
