# Supplementary figures and images for: Prominent crista terminalis mimicking a right atrial mass: A case report and brief review of the literature
Source: Radiol Case Rep. 2021 Dec 9;17(3):434–8. doi: 10.1016/j.radcr.2021.11.028 (PMC8666457; doi:10.1016/j.radcr.2021.11.028)

## Slide 1
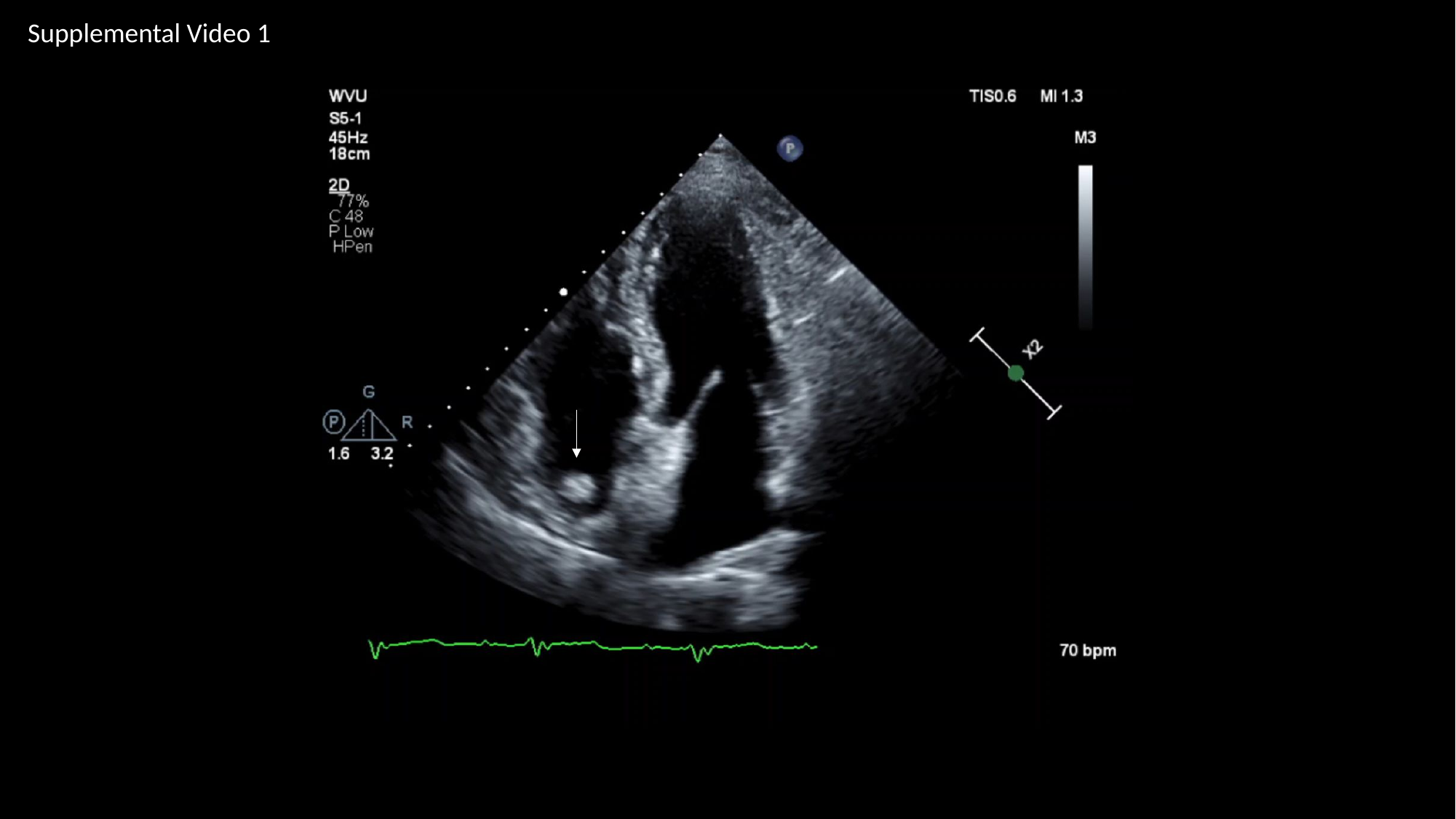

Supplemental Video 1

## Slide 2
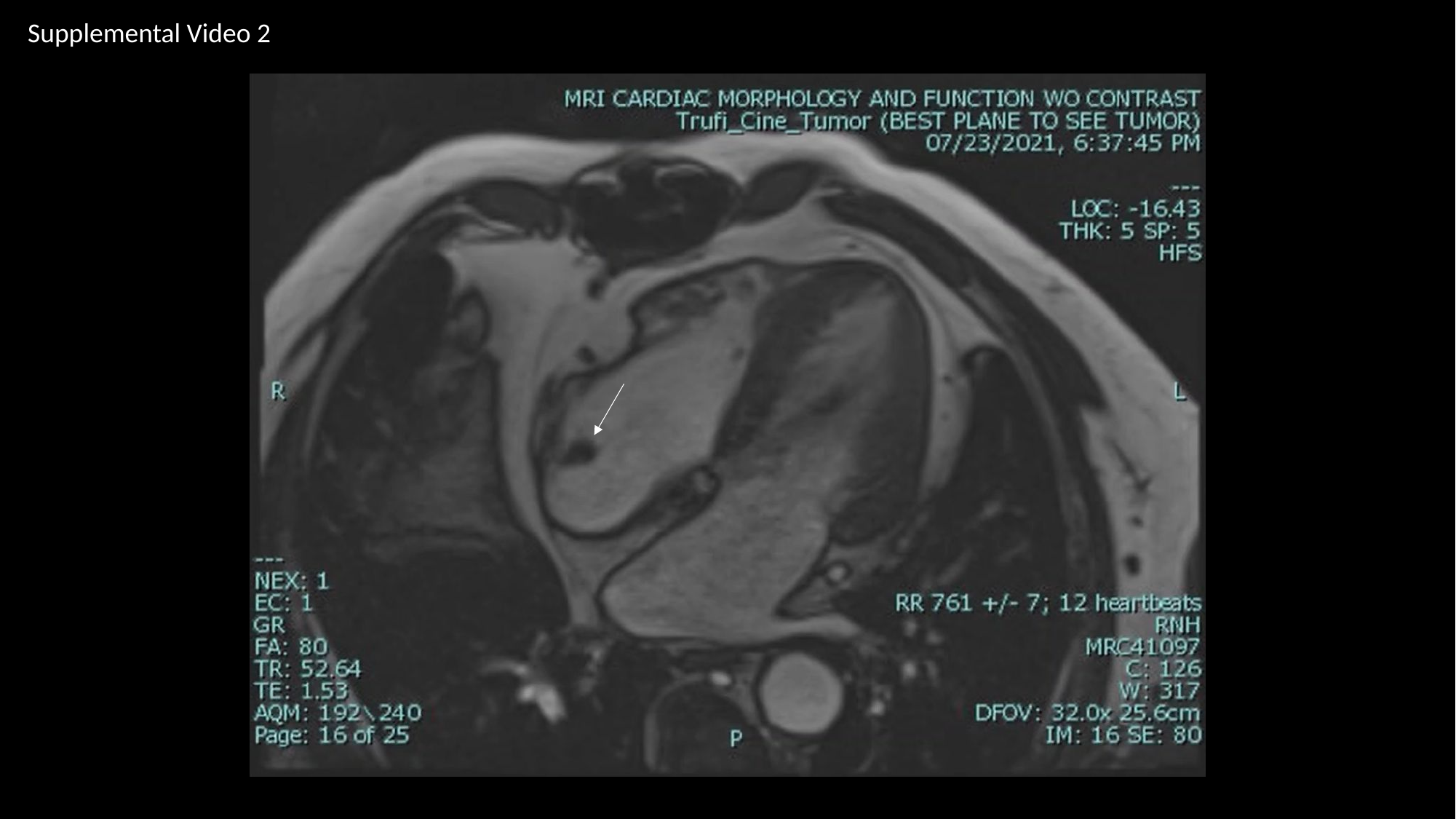

Supplemental Video 2

Supplement: Supplementary file 1 — Supplemental video 1: Transthoracic echocardiogram was performed as a part of preoperative workup prior to surgery. Four-chamber transthoracic echocardiogram shows a prominent crista terminalis (arrow) in the right atrium, which was initially reported as indeterminate, and concerning for malignancy. Further evaluation with CT or MRI cardiac morphology was recommended. Supplemental video 2: Further, evaluation with MRI cardiac morphology was performed. The study was limited due to motion and refusal to administer contrast. TRUFI (true fast imaging with steady-state free precession) Cine images in four-chamber view demonstrates a prominent right atrial intracavitary lesion. [file mmc1.pptx]
